# The Oxidative Fermentation of Ethanol in *Gluconacetobacter diazotrophicus* Is a Two-Step Pathway Catalyzed by a Single Enzyme: Alcohol-Aldehyde Dehydrogenase (ADHa)

**DOI:** 10.3390/ijms16011293

**Published:** 2015-01-07

**Authors:** Saúl Gómez-Manzo, José E. Escamilla, Abigail González-Valdez, Gabriel López-Velázquez, América Vanoye-Carlo, Jaime Marcial-Quino, Ignacio de la Mora-de la Mora, Itzhel Garcia-Torres, Sergio Enríquez-Flores, Martha Lucinda Contreras-Zentella, Roberto Arreguín-Espinosa, Peter M. H. Kroneck, Martha Elena Sosa-Torres

**Affiliations:** 1 Laboratorio de Bioquímica-Genética, Instituto Nacional de Pediatría, S.S. Mexico City 04530, Mexico; E-Mails: glv_1999@yahoo.com (G.L.-V.); america_vc@yahoo.com.mx (A.V.-C.); ignaciodelamora@yahoo.com.mx (I.M.-M.); itzheltorres@hotmail.com (I.G.-T.); sergioenr@gmail.com (S.E.-F.); 2 Departamento de Bioquímica y Biología Estructural, Instituto de Fisiología Celular, Universidad Nacional Autónoma de México, Mexico City 04510, Mexico; E-Mail: mcontre@ifc.unam.mx (M.L.C.-Z.); 3 Departamento de Biología Molecular y Biotecnología, Instituto de Investigaciones Biomédicas, Universidad Nacional Autónoma de México, Mexico City 04510, Mexico; E-Mail: abigaila@correo.biomedicas.unam.mx; 4 Coordinación de Investigación, Facultad de Medicina, Universidad La Salle, Mexico City 14000, Mexico; 5 Cátedras CONACyT, Comisionado a Instituto Nacional de Pediatría, S.S. Mexico City 03940, Mexico; E-Mail: jmarcialqu@conacyt.mx; 6 Departamento de Química de Biomacromoléculas, Instituto de Química, Universidad Nacional Autónoma de México, Circuito Exterior s/n, Ciudad Universitaria, Mexico City 04510, Mexico; E-Mail: arrespin@unam.mx; 7 Fachbereich Biologie, Universität Konstanz, 78457 Konstanz, Germany; E-Mail: Peter.Kroneck@Uni-Konstanz.de; 8 Departamento de Química Inorgánica y Nuclear, Facultad de Química, Universidad Nacional Autónoma de México, Mexico City 04510, Mexico

**Keywords:** bifunctional enzyme-active alcohol dehydrogenase (ADHa), ethanol-acetaldehyde-oxidation, *Gluconacetobacter diazotrophicus*, acetic acid bacteria, alcohol aldehyde dehydrogenase

## Abstract

*Gluconacetobacter diazotrophicus* is a N_2_-fixing bacterium endophyte from sugar cane. The oxidation of ethanol to acetic acid of this organism takes place in the periplasmic space, and this reaction is catalyzed by two membrane-bound enzymes complexes: the alcohol dehydrogenase (ADH) and the aldehyde dehydrogenase (ALDH). We present strong evidence showing that the well-known membrane-bound Alcohol dehydrogenase (ADHa) of *Ga. diazotrophicus* is indeed a double function enzyme, which is able to use primary alcohols (C2–C6) and its respective aldehydes as alternate substrates. Moreover, the enzyme utilizes ethanol as a substrate in a reaction mechanism where this is subjected to a two-step oxidation process to produce acetic acid without releasing the acetaldehyde intermediary to the media. Moreover, we propose a mechanism that, under physiological conditions, might permit a massive conversion of ethanol to acetic acid, as usually occurs in the acetic acid bacteria, but without the transient accumulation of the highly toxic acetaldehyde.

## 1. Introduction

*Gluconacetobacter diazotrophicus* (*Ga. diazotrophicus*) is an aerobic Gram-negative bacterium that belongs to the group of acetic acid bacteria [1]. This group of bacteria is widely distributed in nature and is characterized by their ability to oxidize a variety of sugars and alcohols into organic acids during the fermentation process. It is highly attractive for the food industry because the main characteristic is the conversion of ethanol to acetic acid, an essential component of vinegar, which is produced on a large scale due to its different functions, as an antimicrobial, as well as a conservative and food seasoning [2,3]. In addition this microorganism is rather unique among the acetic acid bacteria because it carries out nitrogen fixation and is a true endophyte originally isolated from sugar cane [4,5,6,7,8], and recently identified in conifers by high-throughput sequencing [9].

Ethanol fermentation by acetic acid bacteria is carried out by two sequential reactions catalyzed by pyrroloquinolinequinone (PQQ)-dependent alcohol dehydrogenase enzymes (ADH) and aldehyde dehydrogenase (ALDH), which are located in the cytoplasmic membrane [6] and transfer electrons to ubiquinone Q10 [7]. PQQ-ADH is a periplasmic quinohemoprotein-cytochrome *c* complex and catalyzes the first step of ethanol oxidation by transferring electrons to Q10 and producing acetaldehyde which usually is the substrate for another enzyme (ALDH), and converted to acetic acid during the second step of ethanol fermentation.

Bacterial alcohol dehydrogenases (ADH) have been classified into three types: Class I ADH (ADH-I) are soluble quinoproteins as in the case of *Pseudomonas aeruginosa* [10] and are similar to the methanol dehydrogenase of methylotrophic bacteria. Class II ADH (ADH-II) are quinohemoproteins widely distributed in Proteobacteria and are expressed as soluble monomers localized in the periplasm, that contain one molecule of PQQ with a single hemo *C* [11,12,13,14]. Class III ADH (ADH-III) are membrane-bound quinohemoproteins described in acetic acid bacteria and localized on the periplasmic side of the cytoplasmic membrane acting as primary dehydrogenases coupled to the respiratory chain via ubiquinone [7]. The substrate specificity of membrane-bound ADH-III of acetic acid bacteria is relatively restricted. However, it has been reported that in some species as *G. polyoxogenes* (formerly *Acetobacter polyoxogenes* [15], *Ga. xylinus* [16], *Acetobacter* sp. SKU 14 [17], *Ga. diazotrophiocus* [18] and *Acetobacter pasteurianus* MSU10 [19], ADH enzymes exhibited activity, also with formaldehyde and acetaldehyde. In previous studies Gomez-Manzo *et al.* [18,20] purified and characterized two types of Class III ADHs from *Ga. diazotrophicus*: ADH active (ADHa) and the inactive (ADHi). Both quinohemoproteins have the same oligomeric and prosthetic group composition. However, both enzymes showed differences in the ability to oxidize alcohol, where the ADHi was several fold less active than its active counterparts [14,20].

In this work, we report the characterization of a double function of the ADHa-III from *Ga. diazotrophicus;* it oxidizes a variety of alcohols and aldehydes and has the ability to oxidize ethanol to acetic acid without releasing the intermediate acetaldehyde into the medium. This activity has not been reported before in any acetic acid bacteria. Our results with the purified ADHa open the possibility to explore the translation of this knowledge to a physiologic context where the mechanism of production of acetic acid by a single enzyme would be of biotechnological interest, because it would prevent the acetification (acetaldehyde production) of culture media compared to other bacteria and have potential for the production of this metabolite in industry. Based on our results we propose a new mechanism with hypothetical intra- and intermolecular pathways in the ADHa heterodimer of *Ga. diazotrophicus*.

## 2. Results

### 2.1. Purification of the ADHa from Ga. diazotrophicus

Previously, we developed a procedure for the purification of the membrane-bound ADH-III of nitrogen-fixing *Ga. diazotrophicus* [18]. We purified the ADHa enzyme following the protocols previously described [18,21]. The purity of the ADHa enzyme was 95% confirmed by SDS-PAGE and corroborated the existence of a heterodimer consisting of two subunits, with molecular masses of ≈72 kDa (S-I) and ≈44 kDa (S-II). UV-vis spectrum confirmed the presence of heme centers in the ADHa enzyme in the reduced state (*i.e.*, 417, 523 and 552 nm, respectively), as previously reported [21]. In the same line, the organic cofactor PQQ of ADHa was identified by electron paramagnetic resonance (EPR). The EPR spectrum of ADHa from *Ga. diazotrophicus* was identical to that previously reported [21] with a well intense narrow line centered at *g* = 2.001, which has been assigned to the PQQ semiquinone radical (PQQsq). A well-resolved resonance pattern with *g*_xyz_ values at 2.003, 1.934, and 1.919 (*g*_av_ 1.953), of the iron-sulfur protein is observed at low temperature (10 K).

### 2.2. N-Terminal Sequence Analysis

To confirm the identity and purity of the isolated ADHa from *Ga. diazotrophicus*, we sequenced two bands from a SDS-PAGE. The *N*-terminal sequence of the subunit-I was digested with trypsin and analyzed by mass spectrometry. The results allowed us to find internal peptides, which were analyzed in the informatic program BLAST, corresponded to the amino acid sequence reported for the S-I of the ADHa (Accession No. YP_001602285). The internal peptides were: (1) YSPLDQINR; (2) GQEGTPLIVDGVMYATTNWSK; (3) VPGNIADK; (4) VYFGTFDGR; (5) VIIGNGGSEFGAR; (6) HVIVHAPK; (7) NGFFYIIDAK; (8) NYVYVNWASGLDPK and (9) DAFYNVVGR. Furthermore, the subunit II was analyzed by using the Ettan MALDI-Tof Pro mass spectrometry, obtaining the following *N*-terminal sequence: MVNRMLNR, which corresponds to the amino acid sequence reported for the S-II of the ADHa (Accession No. YP_001602286). These results suggest strongly that the ADHa purified from *Ga. diazotrophicus* was not contaminated with the ALDH of the same organism.

### 2.3. Kinetic Characterization

To confirm the ability of the pure ADHa enzyme from *Ga. diazotrophicus* to oxidize ethanol and acetaldehyde with high efficiency, we used a coupled assay of the ADHa enzyme with a water-soluble quinone and membranes of *Ga. diazotrophicus* deficient of the ADH enzyme. The activity of the ADHa was followed by measuring the oxidase activity as described in Materials and Methods. The kinetic parameters for both substrates (ethanol and acetaldehyde) were determined employing oxidase activity and compared with the values previously reported [18] for the reductase assay phenazine methosulphate (PMS) plus 2,6-dichlorophenolindophenol (DCPIP). Results for both substrates and both methods are shown in the Table 1. According to these data, the affinity (*K*_m_) of the enzyme ADHa for ethanol in the coupled assay (oxidase activity) was increased one order of magnitude in respect to the dehydrogenase. However, the kinetic constants for acetaldehyde are the same as those found in the reductase and the oxidase assays. As shown in Table 1, the kinetic parameters for the substrate ethanol (*k*_cat_ and *k*_cat_/*K*_m_) obtained in the coupled assays, displayed a better catalysis in respect to the values obtained by the dehydrogenase activity (DCPIP plus PMS). These results show that the membrane-bound ADH has the ability to oxidize both ethanol and acetaldehyde with high efficiency. This also suggests that the water-soluble quinones, such as Q1 and Q2 have a better catalytic efficiency in respect to those artificial acceptors used in standard assays.

**Table 1:** Kinetic constants for the ethanol and acetaldehyde oxidation by ADHa from *Ga. diazotrophicus.*

Kinetic Constants	Ethanol		Acetaldehyde	
	^1^ Dehydrogenase	Oxidase Activity	^1^ Dehydrogenase	Oxidase Activity
*K*_m_ (M)	4.6 × 10^−4^	7.6 × 10^−5^	3.7 × 10^−4^	1.1 × 10^−3^
*k*_cat_ (s^−1^)	1.6 × 10^5^	1.4 × 10^5^	9.3 × 10^4^	1.1 × 10^5^
*k*_cat_/*K*_m_ (M^−1^·s^−1^)	3.6 × 10^8^	1.8 × 10^9^	2.5 × 10^7^	1.0 × 10^8^

^1^ [18].

### 2.4. Thermal Denaturation Curves of the Purified ADHa Enzyme

**Figure 1:**
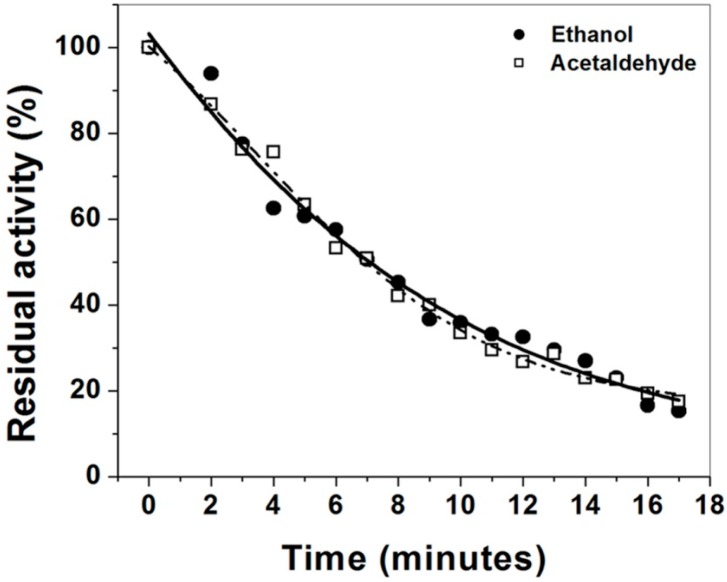
Thermal inactivation of the ADHa. The enzyme was incubated at a constant temperature of 40 °C, and the residual activity was measured at 25 °C for both ethanol and acetaldehyde. The dehydogenase activity was measured under the standard conditions described in Materials and Methods.

The substrate specificity observed in the kinetic constants suggests that the membrane-bound ADHa from *Ga. diazotrophicus* has the ability to oxidize straight-chain alcohols and aldehydes with the same rate. To corroborate that the oxidation of ethanol and acetaldehyde is accomplished by the same ADHa enzyme and was not co-purified with another enzyme we performed thermal denaturation curves incubating the enzyme at 40 °C and the residual activity was measured during this time (Figure 1). The inactivation profile revealed that the ADHa purified from *Ga. diazotrophicus* is fairly thermolabile. Furthermore, the residual activity for ethanol and acetaldehyde were identical, strongly suggesting that ADHa has the ability to oxidized ethanol and acetaldehyde at the same rate, and most probably is a bifunctional enzyme with a similar high efficiency to oxidize ethanol and acetaldehyde.

### 2.5. Determination of Ethanol and Acetate as Final Products of the Reaction

The profile chromatographic separation for the ADHa showed similar activities for both ethanol and acetaldehyde, respectively. In addition to the substrate specificity of the ADHa purified from *Ga. diazotrophicus* [18], the enzyme has the ability to oxidize linear alcohols and aldehydes with great efficiency. We thus performed various assays to determine whether the membrane-bound ADHa purified from *Ga. diazotrophicus* has the ability to oxidize ethanol as a primary substrate and result in acetate as a final product, without releasing the intermediate acetaldehyde into the reaction medium.

#### 2.5.1. Gas Chromatography-Mass Spectrometry (GC-MS)

**Figure 2:**
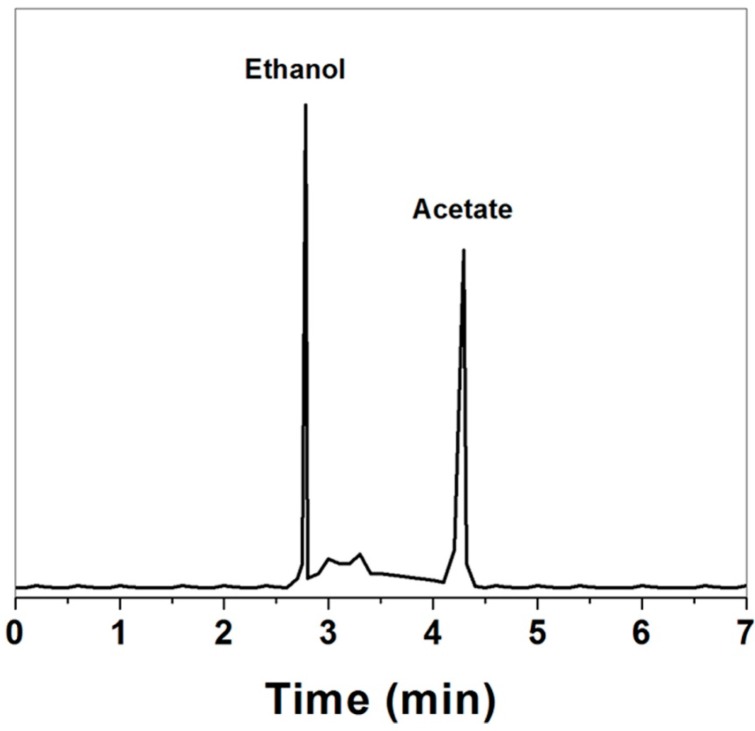
Chromatogram obtained from the analysis of the products in the final reaction by GC-MS. The purified ADHa enzyme from *Ga. diazotrophicus* was suspended in potassium phosphate buffer, pH 6.0. Potassium ferricyanide was used as an electron acceptor, and ethanol was used as the initial substrate.

The purified ADHa enzyme was incubated with potassium ferricyanide as an electron acceptor and ethanol was used as an initial substrate in a hermetically closed tube. After the reaction was completed, an aliquot of the final reaction mixture was injected in the JEOL/JM-AXSOSHA instrument, previously equilibrated. The reaction products are shown in Figure 2. There we observe a mixture of ethanol (initial substrate) and the acetate (final product) with retention time of 2.73 and 4.29 min, respectively. However, we do not observe the presence of acetaldehyde (1.66 min) as a final product of the reaction catalyzed by the enzyme ADHa. The retention times in the final reaction mixture are in agreement with those obtained for ethanol and acetate, used as standards.

#### 2.5.2. Nuclear Magnetic Resonance (NMR)

**Figure 3:**
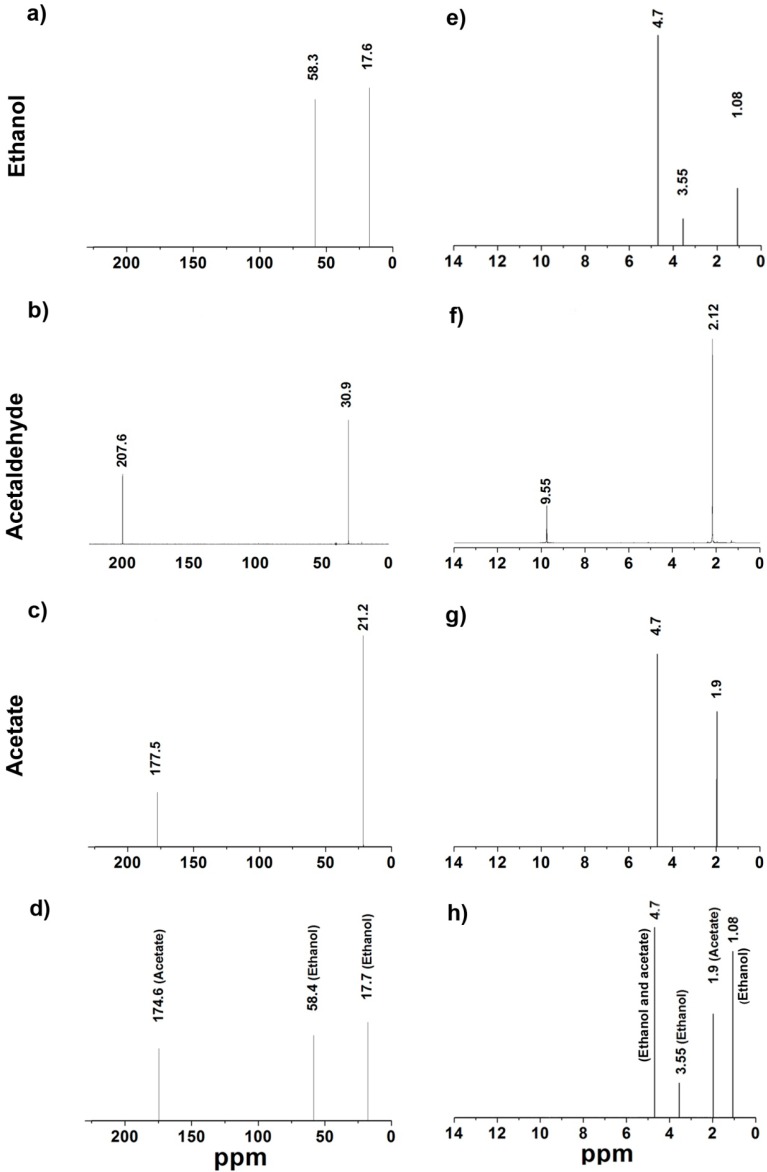
NMR of the compounds contained in the final reaction mixture catalyzed by the ADHa of *Ga. diazotrophicus*. (**a**) The ^13^C signals obtained for the standard ethanol; (**b**) acetaldehyde; (**c**) acetate and (**d**) the final products catalyzed by the ADHa enzyme; (**e**) The ^1^H signals for ethanol; (**f**) acetaldehyde; (**g**) acetate and (**h**) the final products catalyzed by the ADHa. The ^13^C and the ^1^H of ethanol, acetaldehyde and acetate suspended in deuterated water were also determined.

NMR was used as an unambiguous technique to determine the nature of the final products in the catalytic activity of ADHa; we observed the ^1^H and ^13^C signals of the compounds present in the final reaction mixture in a hermetically closed tube. Thus, the ^13^C signal obtained for the standard ethanol, acetaldehyde, and acetate are showed in the Figure 3a–c, respectively. The ^1^H signals for ethanol, acetaldehyde and acetate are showed in the Figure 3e–g, respectively. Then, the signals obtained for the final reaction mixture catalyzed by the ADHa for ^13^C and ^1^H are shown in Figure 3d,h, respectively. The ^13^C signals at 17.8 and 58.4 found in the final reaction mixture correspond to the signals of ethanol (in excess); the signal at 174.7 ppm corresponds to one of the signals of the acetate. The ^1^H signals at 1.08 and 3.55 and 4.7 ppm correspond to the signals for ethanol, and the signals of the reaction mixture at 1.9 and 4.7 correspond to the signals of the acetate. Therefore, since there are no acetaldehyde signals detected as a final product of the reaction, acetate was the only product detected in the final reaction of the enzyme ADHa. These results also suggest that the acetate is the final product of the reaction catalyzed and that the fermentative oxidation of ethanol in *Ga. diazotrophicus* is a two-step pathway catalyzed by a single enzyme: Alcohol-Aldehyde dehydrogenase (ADHa).

#### 2.5.3. Acetaldehyde-Semicarbazone Formation with Radiolabeled Ethanol

Another assay used to measure the final product of the enzyme ADHa was the acetaldehyde-carbazide method employing radioactive [1-^14^C]ethanol as initial substrate, and after the ferricyanide reduction, the radioactivity [1-^14^C]ethanol in counts per minute (CPM) was measured. If the ADHa enzyme itself produces acetaldehyde and releases it to the reaction medium, the semicarbazide must react with the acetaldehyde and form the acetaldehyde-semicarbazone complex, which would cause a shift in the signal of semicarbazide (204 nm) to semicarbazone (223 nm). In the same order, the radioactivity will be present in the complex semicarbazone-acetaldehyde. The results obtained are shown in Table 2.

**Table 2:** Acetaldehyde-semicarbazone formation with [1-^14^C]ethanol radiolabeled.

Assay	Acetaldehyde-Semicarbazone Complex (CPM)	Final Reaction Mixture Catalyzed by the ADHa (CPM)
Control (−)	13,285	386,175
Control (+) (ADH-NADH)	89,640	310,360
ADHa	12,525	383,400

CPM: Counts per minute.

The semicarbazide present in the negative control showed a radioactivity [1-^14^C]ethanol of 13,285 CPM; this radioactivity probably due to ethanol evaporation. As positive control, we used the ADH enzyme dependent of NAD^+^, which has the ability to oxidize the ethanol to acetaldehyde and releasing to the reaction medium. As showed in the Table 2, the positive control showed a radioactivity [1-^14^C]ethanol of 89,640 CPM in the semicarbazide solution. However, the semicarbazide present in the reaction of the ADHa enzyme showed a radioactivity [1-^14^C]ethanol of 12,525 CPM. The higher radioactivity was present in the reaction mixture (383,400 CPM), which corresponds at the initial radioactivity (100%), assuming that the amount of radioactivity determined in the semicarbazide solution is due to ethanol evaporation as was observed in the negative control. These data suggest again that the enzyme ADHa does not release the acetaldehyde to the reaction medium and by itself has the ability to oxidize ethanol to acetate without the intervention of the membrane-bound ALDH enzyme from *Ga. diazotrophicus*.

**Figure 4:**
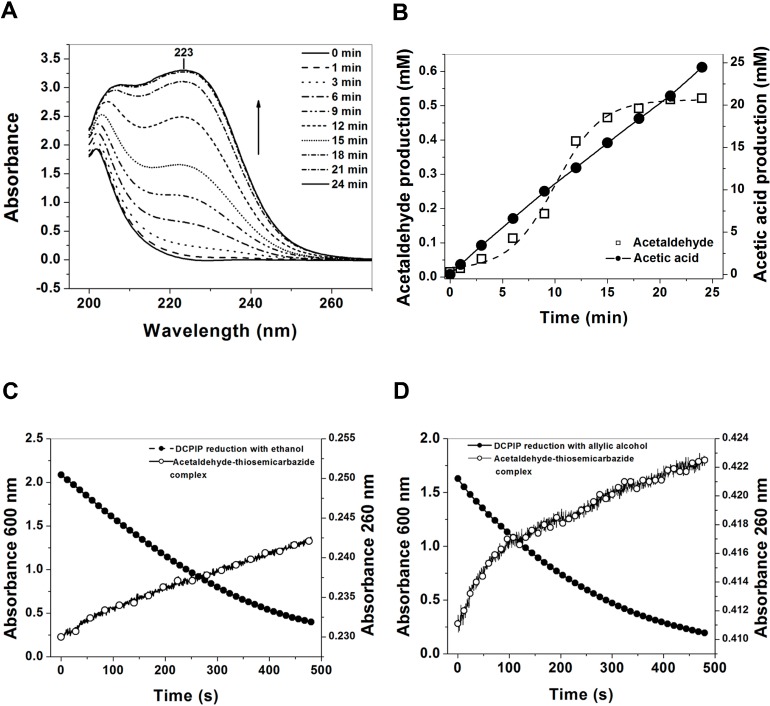
Determination of acetaldehyde-semicarbazone complex as the final product of the ADHa. (**A**) UV/vis spectra of the formation of acetaldehyde-semicarbazone complex at 223 nm. Spectra was recorded at three min intervals; (**B**) Determination of the acetaldehyde released during the reaction by the enzyme ADHa and trapped by the semicarbazide; (**C**) Time course of ethanol and (**D**) allylic alcohol oxidation by the ADHa from *Ga. diazotrophicus*.

To corroborate the results obtained using radioactive [1-^14^C]ethanol, the same test was performed under the same experimental conditions using ethanol HPLC grade. The formation of the acetaldehyde-semicarbazone complex was measured during time in hermetically closed cuvettes (Figure 4A). As seen, the absorbance spectrum of the semicarbazide showed a shift in signal from semicarbazide to semicarbazone (223 nm) as showed in the Figure 4A. According to the signal intensity and using the calibration curve with acetaldehyde-semicarbazone, we determined that the concentration of acetaldehyde released during the reaction by the enzyme ADHa and trapped by the semicarbazide was 0.5 mM (Figure 4B); which corresponds to 2.3% of the final product, when the reaction was started with an excess of ethanol (100 mM). However, if we consider the specificity constant (*k*cat) and the reaction time in this assay, we calculate that of the 100 mM of initial substrate, only 25 mM ethanol has been catalyzed to product by ADHa in 24 min. Thus, from 100% of product (25 mM), 97.3% of the oxidized ethanol is brought to acetate form and only 2% (0.5 mM) is released as acetaldehyde.

#### 2.5.4. Coupled Assay of 2,6-Dichlorophenolindophenol (DCPIP) with Semicarbazide and Thiosemicarbazide

Finally, we designed an assay where two reaction cells were coupled, connected by a latex tube and hermetically closed, which allowed us to continuously measure the DCPIP reduction and the absorbance change at 260 nm simultaneously, when the thiosemicarbazide reacts with the acetaldehyde to form the acetaldehyde-thiosemicarbazone complex [22]. Under the same test conditions, we changed the semicarbazide by thiosemicarbazide, due to the latter having a greater sensitivity for acetaldehyde [22]. It is observed that at initial velocities obtained with DCPIP the concentration of oxidized ethanol was 16.9 mmol of substrate min^−1^·mg^−1^ (Figure 4C). Therefore, the increase in absorbance at 260 nm was followed over time (Figure 4C), which indicates the formation of acetaldehyde-thiosemicarbazone complex. Using the signal intensity and according to the calibration curve with acetaldehyde-thiosemicarbazide complex, we determined that the concentration of acetaldehyde released during the reaction and trapped by the thiosemicarbazide to form thiosemicarbazone was 0.3 mM. Based on these data, we concluded that from the 100% oxidized ethanol by the ADHa enzyme, only 1.8% of the ethanol is oxidized in the form of acetaldehyde (0.3 mM thiosemicarbazone) is released to the reaction medium, and that 98% of the substrate is carried to acetate (16.9 mM of substrate min^−1^·mg^−1^). Furthermore, we performed the same assay with allylic alcohol as the initial substrate and observed that from the 100% allylic alcohol oxidized by the ADHa enzyme, only 2.9% of the respective aldehyde is released to the reaction medium, and 97.4% of the substrate is carried to acetate (Figure 4D). These results leads us to conclude that the ADHa from *Ga. diazotrophicus* is able to perform double oxidation of ethanol to acetate without releasing significant quantities of acetaldehyde to the reaction medium.

## 3. Discussion

The ADHs of the acetic acid bacteria function as primary dehydrogenases in the respiratory chain, where electrons removed by the enzyme are initially transferred to the prosthetic group PQQ and subsequently passed to four *c*-type cytochromes present in the enzyme (one located in the SU-I *cI* and three located on the SU-II, *cII1*, *cII2* and *cII3*) and the ubiquinone (UQ9-10).

The substrate specificity of purified ADHs of acetic acid bacteria has been tested only in a few cases, e.g., the ADHs of *A. aceti* [23] and *G. suboxidans* [23,24] cannot oxidize aldehydes at all. On the other hand, the ADH of *G. polyoxogenes* showed 60% and 18% of relative activity on formaldehyde and acetaldehyde respectively, as compared with ethanol activity [15] and the ADH of *Ga. xylinus* showed 13% and 34% of relative activity on acetaldehyde and formaldehyde respect to ethanol using the method of ferricyanide reductase [16]. Furthermore, Kanchanarach *et al.* [19] reported that the ADH of *A. pasteurianus* MSU10 showed 63% and 78% of relative activity on formaldehyde and acetaldehyde respectively, as compared with ethanol activity. Interestingly, it was previously reported that the ADH-III of *G. polyoxogenes* (formerly *Acetobacter polyoxogenes* [15], *Ga. xylinus* [16], *Acetobacter* sp. SKU 14 [17], *Ga. diazotrophiocus* [18] and *Acetobacter pasteurianus* MSU10 [19], showed significant activity on formaldehyde and acetaldehyde, respectively.

Gómez-Manzo and co-workers [18] reported that ADHa purified enzyme from *Ga. diazotrophicus* has the ability to oxidize linear alcohols as Ethanol, *n*-Propanol and *n*-Butanol and aldehydes with almost the same rate. It is important to note that the affinity of ADHa (*K*_m_ = 4.6 × 10^−4^ M) to acetaldehyde substrate compared with the membrane-bound ALDH (*K*_m_ = 3.3 × 10^−3^ M) from the same organism [25] is of order higher. Additionally, the purified ADHa from *Ga. diazotrophicus* showed a better affinity in respect to the membrane-bound ALDH from *A. aceti* (*i.e.*, 2.9 × 10^−3^ M [26]), *G. suboxydans* (*i.e.*, 3.3 × 10^−3^ M [27]) and *A. rances* (*i.e.*, 1.0 × 10^−3^ M [28]. This suggests that ADHa captures acetaldehyde at low concentrations, promoting its rapid oxidation to acetic acid. For these reason we decided to perform assays to determine whether the enzyme ADHa membranous (ADH-III) itself, purified from *Ga. diazotrophicus* has the ability to oxidize ethanol as the primary substrate and produce acetate as a final product, without releasing the intermediate acetaldehyde to the reaction medium. Different techniques such GC-MS, NMR, acetaldehyde-semicarbazone complex formation with radiolabeled ethanol and coupled assay of DCPIP with thiosemicarbazide demonstrated that the ADHa enzyme is capable of oxidizing ethanol to acetate without intervention of the enzyme ALDH. This property indicates the possibility of an ADHa as a bifunctional enzyme in terms of its ability to oxidize alcohols and aldehydes with high efficiency, contrary to that which occurs in the NAD^+^ dependent bifunctional ADHs. These latter molecules appear to be a fusion product of two genes (ADH and ALDH) joined by a linker sequence; the *N*-terminus is highly homologous to the family of NAD^+^ dependent aldehydes oxidoreductases, and the *C*-terminus is homologous to the family of NAD^+^ dependent alcohol oxidoreductases that requires Fe^2+^ as cofactor. These ADH enzymes have been described in *Clostridium acetobutylicum* [29], *Escherichia coli* [30,31], *Polytomella* sp [32] and *Leuconostoc mesenteroides* [33].

PQQ dependent enzymes function as a prosthetic group, and only the quinohemoprotein ADH-II has been characterized, and corresponding to the *Comamonas testosteroni* (formerly *Pseudomonas testosteroni*). This enzyme has the ability to oxidize a wide range of primary alcohols and aldehydes [12]. Although this enzyme oxidizes alcohols and aldehydes, it was demonstrated that the enzyme alone is capable of oxidizing ethanol, and the acetaldehyde is released as an intermediary in the reaction medium.

**Figure 5:**
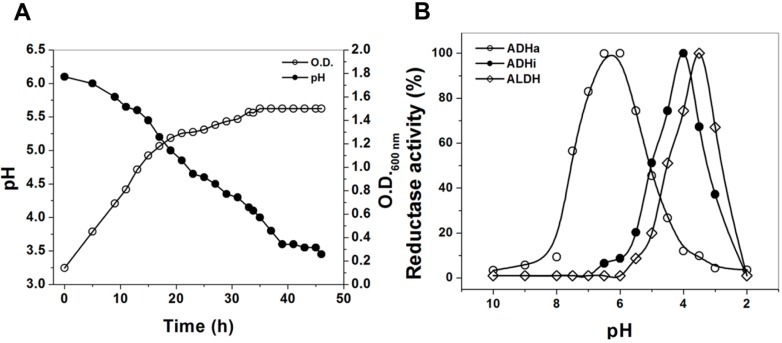
(**A**) Growth properties of *Ga. diazotrophicus* in LGIP medium. (○) Determination of growth by optical density O.D._600nm_ and (●) Measurement of pH of the culture medium; (**B**) Quantification of reductase activity pH dependent properties of the purified membrane-bound Alcohol dehydrogenase active (ADHa), Alcohol dehydrogenase inactive (ADHi) and aldehyde dehydrogenase (ALDH) complexes purified from *Ga. diazotrophicus*. The ferricyanide reductase activity was measured in Mcllvaine buffer at different pH. The conditions used for the activity assays are described in Experimental Setion.

Finally, taking into account the optimum pH previously reported for the membrane-bound active alcohol dehydrogenase (ADHa) [18], inactive alcohol dehydrogenase (ADHi; 15% of activity in respect to the active ADH) [20], and aldehyde dehydrogenase (ALDH) [25] from *Ga. diazotrophicus*, we propose that under physiological conditions, the bifunctional ADHa would permit the massive conversion of ethanol to acetic acid, usually seen in the acetic acid bacteria, without the inconvenient transient accumulation of the highly toxic acetaldehyde. Our results suggest that at the beginning of the growth of *Ga. diazoptrophicus* (the first 5 to 10 h; Figure 5A), the ADHa with an optimum pH 6.0 (Figure 5B), might be able perform a rapid oxidation from ethanol to acetic acid present in the medium, and that these substrates are subjected to a two-step oxidation to produce acetic acid without releasing the acetaldehyde intermediary to the media. At the end phase of the growth (pH 3.5, after 30 to 40 h; Figure 5A) the ADHi, with an optimum pH of 4 [20] might be able to oxidize the small quantity of alcohol remaining in the culture medium, and the ALDH (optimum pH 3.5) would convert the acetaldehyde released in the media to acetate (Figure 5B). These data are in concordance with the aldehyde-ferricyanide reductase activity in native membranes of *Ga. diazotrophicus* which exhibited an optimum pH of 3.5 [25]. Therefore the results here indicate that the optimal pH determined for the ferricyanide reductase activity both in membranes and purified ALDH enzyme [25] is similar to the pH range at which acetic acid bacteria usually produce vinegar.

**Figure 6:**
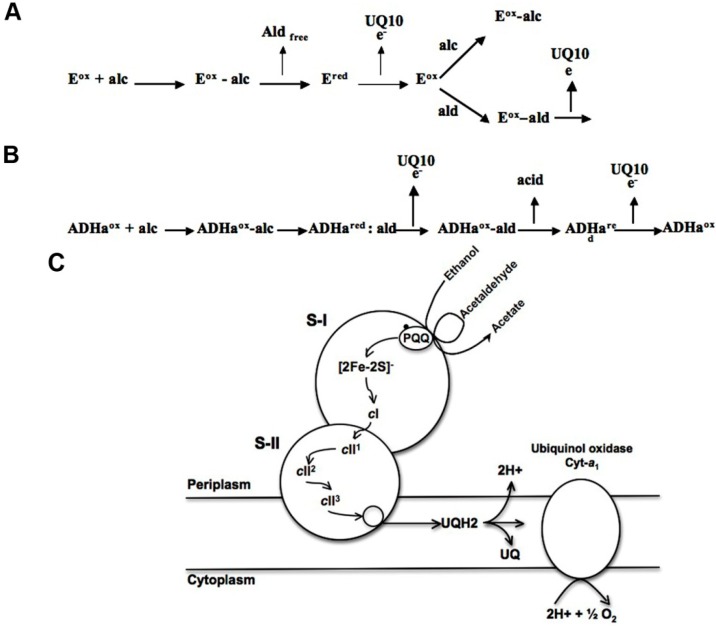
Proposal of the mechanism for the oxidation of ethanol by the ADH-II and ADH-III enzymes. (**A**) Reaction mechanism proposed for ADH-II from *Comamonas testosteroni* [13]. E^ox^ = oxidized enzyme. E^red^ = reduced enzyme. UQ10 = endogenous ubiquinone 10. alc = alcohol. ald = aldehyde; (**B**) Alternative mechanism proposed for ADHa-III from *Ga. diazotrophicus*. ADHa^ox^ = ADHa oxidized. ADHa^red^ = ADHa reduced. UQ10 = endogenous ubiquinone-10. alc = alcohol. ald = aldehyde; and (**C**) Hypothetical intra- and intermolecular electron transfer pathways in the heterodimeric membrane-bound ADHa of *Ga. diazotrophicus*. PQQ, [2Fe–2S], and cytochrome *cI* are assigned to SUI and cytochromes *cII1*, *cII2*, and *cII3* to SUII, interacting with UQ10.

Our results substantially differ with the reaction mechanism proposed for the ADH enzymes in acetic acid bacteria, for example the ADH-II from *Comamonas testosteroni* [13] that releases the intermediate acetaldehyde into the medium and then this compound competes for the active site as an alternative mechanism (Figure 6A). In our case there is no competition of the intermediate acetaldehyde due to the fact that the latter is not released at the active site (double sequential reduction) as shown in the Figure 6B.

## 4. Experimental Section

### 4.1. Strain and Growth Conditions

*Ga. diazotrophicus* PAL5 (ATCC 49037) was grown at 30 °C in a modified version of LGI medium (LGIP medium), under the conditions described by Reis *et al.* [34], in a 60-L-working-volume Bioflow 5000 fermenter (New Brunswick Scientific, NJ, USA), stirred at 120 rpm and aerated at 60-L of air·min^−1^. Cells were harvested in the early stationary phase (48 h). Procedures used for the disruption of cells and membrane preparation were described earlier [35]. The membranes were frozen and stored in liquid nitrogen without significant loss of enzymatic respiratory activities.

### 4.2. Purification of the ADHa from Ga. diazotrophicus

The enzyme was removed from the membranal fraction with Triton X-100 (0.5%). The ADHa enzyme was purified according to Gómez-Manzo *et al.* [18]. All operations were performed in 10 mM phosphate, pH 6.0, containing 0.1% Triton X-100. The purity was checked by SDS-PAGE (16 × 14 cm slab gels), 10% polyacrylamide [36] and were stained with 0.05% Coomassie brilliant blue R-250 [24]. The protein concentration was determined by a modified Lowry procedure [37] with bovine serum albumin as standard.

### 4.3. Spectroscopy Studies

The purified enzyme was suspended in standard buffer and examined with a Shimadzu UV-2401 PC spectrophotometer, using cuvettes with a 1-mm light path. Heme *C* of ADH was spectroscopically identified from its absorption spectrum at 25 °C as described by Escamilla *et al.* [35]. The presence of reduced PQQ in the purified ADHa was confirmed from its absorption spectrum (maximum peak at 352 nm), as reported previously for other PQQ-containing preparations [38]. Furthermore, purified ADH (13 mg total protein) in 20 mM potassium phosphate, pH 6.0, was transferred into a quartz tube and frozen. The CW-EPR spectra were recorded on a Bruker Elexsys E500 spectrometer with a X-band (9.38 GHz), and 100-kHz modulation [21]. The temperature at 10 K was maintained with an Oxford liquid Helium continuous flow cryostat (Bruker, Billerica, MA, USA).

### 4.4. Thermal Inactivation of the Purified Enzyme ADHa

Thermal inactivation curves of the purified ADH were performed with ethanol or acetaldehyde as substrates, in independent experiments. The residual activity of both ethanol and acetaldehyde were tested at a concentration of 20 mM, and PMS plus DCPIP as carrier and electron acceptors were used [24]. The enzyme (1 mg/mL) was incubated at a constant temperature of 40 °C, and an aliquot was taken each minute during 17 min and the enzymatic activity was measured at 25 °C for both ethanol and acetaldehyde.

### 4.5. Enzymatic Activity Assays

The dehydrogenase activities associated to membranes and purified fractions were determined by spectrophotometry using potassium ferricyanide as the electron acceptor according to the standard method described by Matsushita *et al.* [8], or with PMS plus DCPIP as electron acceptors [18,24]. A pH of 6.0 was used routinely. Initial velocity data were obtained by varying the substrate concentrations ranging from 0 to 5 mM. Ethanol and acetaldehyde and other substrates tested were used at a concentration of 20 mM. The kinetic parameters *V*_max_ and *K*_m_ were calculated by fitting initial velocity data to the Michaelis-Menten equation (*v_i_* = *V*_max_[S]/*K*_m_ + [S]). Oxidase activities were determined at 30 °C with a Clark oxygen electrode using a 53YSI oxygen meter. Membranes (0.1 mg protein) were treated previously with 1% Triton X-100 and suspended in a final volume of 2 mL of 10 mM potassium phosphate buffer, pH 6.0. This mixture was added with 30 μg purified ADHa of *Ga. diazotrophicus*, 10 mM ethanol or acetaldehyde. The reaction was started with the addition of quinone analogous at final concentration of 100 μM. The quinone analogous used were: Q1 or Q2. All the determinations were carried at 30 °C. The values for dehydrogenases and oxidase activity are the average of three independent experiments; in all the cases, standard errors were less than 5%. Enzyme reaction was carried out in the presence of 0.1% Triton X-100.

### 4.6. Determination of Ethanol and Acetate as Final Products of the Reaction

#### 4.6.1. Gas Chromatography-Mass Spectrometry (GC-MS)

The enzyme ADH (1 mg/mL) purified from *Ga. diazotrophicus* was suspended in 0.01 M potassium phosphate buffer, pH 6.0. Furthermore, 0.025 M potassium ferricyanide was used as an electron acceptor and 0.1 M ethanol was used as the initial substrate. The reaction was carried out in a hermetically closed tube with a final volume of 1 mL of reaction. After the total reduction of potassium ferricyanide (from yellow to colourless), an aliquot of the final reaction mixture was injected in the JEOL/JM-AXSOSHA instrument, which was previously equilibrated. Ethanol, acetaldehyde and acetate (0.1 M) were used as standards, respectively.

#### 4.6.2. Nuclear Magnetic Resonance (NMR)

^1^H and ^13^C NMR from the pure ethanol, acetaldehyde and acetic acid were recorded as standards on a Varian NMR Unity Plus 500 spectrometer equipped with an Oxford cryostat. The work frequencies for ^13^C and ^1^H NMR were 500 and 250 MHz respectively. The reaction mixture was studied under the same conditions as previously described for GC-MS, and was carried out in a hermetically closed tube with a final volume of 1 mL of reaction. Both the mixture reaction and the standards were suspended in deuterated water.

#### 4.6.3. Acetaldehyde-Semicarbazone Formation with Radiolabeled Ethanol

An alternative method to characterize the final products of the catalyzed oxidation of ethanol by ADH, was the assay of the acetaldehyde-semicarbazone formation with radiolabeled [1-^14^C]ethanol. This assay was performed in 50 mL flasks hermetically closed containing a support for fixing a 1.6 mL Eppendorf tube, containing 300 µL of 0.05 M of semicarbazide and which was not in contact with the reaction mixture. The semicarbazide reagent was prepared in 0.01 M sodium phosphate buffer, pH 6.0. The ADH enzyme (1 mg/mL) was suspended in 0.01 M potassium phosphate buffer, pH 6.0. Then, 0.02 M Potassium ferricyanide was added as an electron acceptor and 0.1 M [1-^14^C]ethanol was used as initial substrate. After the total reduction of potassium ferricyanide (from yellow to colourless), the radioactivity of the [1-^14^C]ethanol was measured both in the reaction mixture and in the solution that contained the semicarbazide (Eppendorf tube). As a negative control (no ADHa enzyme) a 0.01 M phosphate buffer solution pH = 6 0, 0.02 M potassium ferricyanide as an electron acceptor and 0.1 M of radioactive [1-^14^C]ethanol as initial substrate was used. As a positive control the enzyme NADH-dependent alcohol dehydrogenase (obtained from Sigma Co., San Luis, MO, USA); which has the ability to oxidize the ethanol and release the acetaldehyde as final product of the reaction was used.

#### 4.6.4. Coupled Assay of 2,6-Dichlorophenolindophenol (DCPIP) with Carbazide and Thiosemicarbazide

We designed an assay in which two reaction cells were coupled, to measure continuously the reduction of DCPIP plus PMS as electron acceptors [24], in order to measure the absorbance changes at 223 nm for the acetaldehyde-carbazide complex and the formation the acetaldehyde-thiosemicarbazone complex at 260 nm (reaction between the thiosemicarbazide and acetaldehyde), simultaneously [22].

Both assays were accomplished in two quartz cells (1 mL) which were connected by a latex tube and hermetically closed. The first cell contained the ADHa enzyme (300 µg) suspended in 0.01 M potassium phosphate buffer pH 6.0, 0.08 M DCPIP as electron acceptor and 0.06 M PMS as electron mediator plus the 0.02 M ethanol as initial substrate. The second cell contained 300 µL of 0.05 M carbazide or thiosemicarbazide solution in the same buffer, where the carbazide or thiosemicarbazide reacted with acetaldehyde to form a specific acetaldehyde-carbazide or acetaldehyde-thiosemicarbazone complexes with a maximum absorbance at 223 and 260 nm, respectively. The negative control was performed without enzyme, and as positive control, acetaldehyde HPLC grade was used. Due to lack of a known molar extinction coefficient (ε) for acetaldehyde-carbazone or acetaldehyde-thiosemicarbazone complexes, we performed a calibration curve with known concentrations of acetaldehyde. Thereby, we were able to quantify the concentration of acetaldehyde released during the reaction. It is interesting to note that the thiosemicarbazide method has a sensitivity of 2.2 × 10^−6^ M for acetaldehyde according to reports from Djurdjic and Stojanovic [22].

## 5. Conclusions

In conclusion, in this work we demonstrated new evidence using different experimental approaches that the oxidative fermentation of ethanol in *Ga. diazotrophicus* follows a different mechanism than other acid-acetic bacterium that involves a two-step pathway catalyzed by a single enzyme: Alcohol-Aldehyde dehydrogenase (ADHa). Our results confirm that the ADHa enzyme has the ability to oxidize alcohols as well as aldehydes, and utilizes a reaction mechanism where the substrate ethanol is subjected to a two-step oxidation to produce acetic acid without releasing the acetaldehyde intermediary into the media (Figure 6C). In a physiological context, this enzyme allows the growth of *Ga. diazotrophicus* at pH 6 avoiding acetaldehyde toxicity; when pH falls below 6 by acetate accumulation, the aldehyde dehydrogenase (ALDH) becomes active. Additionally, these enzyme results are highly attractive for biotechnological processes for simplifying the steps of production of metabolites of interest without acetaldehyde accumulation. The ADHa provides an advantage over other acetic acid bacteria which require two different enzymes to transform ethanol and generate other final products.
